# Evaluation of changes in cytochrome P450 2C19 activity in type 2 diabetic rats before and after treatment, by using isolated perfused liver model

**DOI:** 10.22038/ijbms.2020.40836.9642

**Published:** 2020-05

**Authors:** Navid Neyshaburinezhad, Mohammad Reza Rouini, Hanieh Entezari, Hoda Lavasani, Yalda Hosseinzadeh Ardakani

**Affiliations:** 1 Biopharmaceutics and Pharmacokinetic Division, Department of Pharmaceutics, Faculty of Pharmacy, Tehran University of Medical Sciences, Tehran, Iran

**Keywords:** CYP2C19, Cinnamon, Isolated hepatic perfusion, Metformin, Phenoconversion, Type 2 diabetes

## Abstract

**Objective(s):**

Alteration in drug metabolism is very likely in diabetes mellitus. This study assessed changes in CYP2C19 enzymatic activity in the liver using omeprazole as a probe in the animal model of type II diabetes (T2DM) before and after treatment with metformin and cinnamon.

**Materials and Methods:**

Twenty-eight male Wistar rats were randomly divided into seven groups. Fourteen days after induction of type 2 diabetic mellitus (T2DM), rats in the test group received metformin, cinnamon, and metformin plus cinnamon daily for 14 days. On day 28, rats were subjected to liver perfusion by Krebs-Henseleit buffer containing omeprazole as a CYP2C19 probe. Perfusate samples were analyzed by HPLC-UV to evaluate the activity of CYP2C19.

**Results:**

Mean metabolic ratio of omeprazole was changed from 0.091±0.005 in the control group to 0.054±0.005 in the untreated-diabetic rats. This average was increased inordinately to 0.218±0.036 in the treated rats with metformin. Interestingly, the administration of cinnamon in combination with metformin in diabetic rats caused the enzyme activity to return to (0.085±0.002) approximately the observed levels in the control group (0.091±0.005).

**Conclusion:**

Results showed that despite the suppression of the CYP2C19 enzyme activity in T2DM rats, metformin treatment could increase the enzyme activity. Simultaneous application of cinnamon and metformin can modulate the function of CYP2C19 to the observed level in the control group and make it more predictable to treat diabetes mellitus and fate of drugs that are metabolized by this enzyme.

## Introduction

The monooxygenase systems are major active enzymes in phase I reactions. In these reactions, substrate encounters oxidation, reduction, hydrolysis, hydration, and several other reactions (1). Although cytochromes P450 (CYPs) are divided into 18 families and 57 subfamilies, the most important CYPs with the largest contribution in xenobiotic metabolisms are CYP3A4, CYP2D6, CYP2C9, CYP2C19, and CYP1A2, respectively (2). Meanwhile, CYP2C19 is involved in the metabolism of at least 10% of the medications present in the clinic, including antidepressants, proton pump inhibitors (PPIs), anticoagulants, anticonvulsants, anti-malarias, and anxiolytics (3, 4). It became known that changing levels of cytochrome P450 enzymes can considerably affect the pharmacokinetics of medications and clinical response (5, 6). This would be more important in medications with a narrow therapeutic index or prodrugs requiring metabolizing systems for activation (7). Genetic polymorphism is one of the most critical features of CYP2C19. This enzyme shows deficient activity in 3-5% of the Caucasian and 15-20% of the Asian population (8, 9).

Furthermore, the expression of most genes in the family of CYP1, CYP2, CYP3, and CYP4 could be induced or inhibited by multiple environmental factors. Some studies have reported that certain, acute or chronic diseases can affect the levels of cytochrome P450 enzymes by altering gene expression through stimulating the inflammatory response (10). A wide range of diseases, including cancers, microbial infections, rheumatoid arthritis, head trauma, and some cardiovascular diseases can stimulate inflammatory responses in the body (11, 12). Type 2 diabetic mellitus (T2DM) is a severe metabolic disorder that can decrease the quality of life, if not treated properly. Considering the nature of diabetes and various vascular (microvascular and macrovascular) and non-vascular disorders, changes in the absorption, distribution, and metabolism of drugs is highly probable (13, 14). Several studies have investigated the content of liver microsomal enzymes in diabetic patients in comparison with healthy controls (15, 16). As previously mentioned, most studies have merely focused on protein content and mRNA expression as well as variations in the metabolic ratio of antipyrine as a probe for the CYP450 activity (17-19). Considering the high prevalence of diabetes along with the use of various medications besides drugs associated with controlling blood glucose levels in these patients, a high priority has been given to the study of the effect of diabetes as well as its physiological and pathological changes on the pharmacokinetics of drugs. Thus, it is very important to evaluate diabetes-induced changes in the activity of CYP2C19, as one of the most important metabolic enzymes. On the other hand, the use of herbal medicines has increased dramatically over the past few decades. Regardless of their increasing applications, there are some concerns about their impact on other medications and treatment processes (20). It should be noted that the simultaneous use of drugs and herbal supplements can lead to new and unexpected interactions due to changes in drug pharmacokinetics (21, 22). 

Additionally, cinnamon is generally used as a traditional medicine in many countries to reduce blood glucose levels in diabetic patients (23, 24). Although recent studies have demonstrated its anti-inflammatory and hypoglycemic effects and its effects on improving metabolic syndrome (24-26), its potential effects on other common processes in the body, such as the expression of carriers and enzymes, have rarely been taken into consideration. Moreover, many patients do not disclose the use of herbal supplements to their physician. This is expected to be one of the main reasons for the failure of therapeutic responses (27).

Based on the above, an animal model of diabetes was considered in this study to investigate the effect of diabetes and the potential impact of cinnamon on the CYP2C19 activity. Since the streptozotocin (STZ)-induced diabetes was widely used in pharmacokinetic studies, the same approach was chosen in this study.

On the other hand, liver perfusion has been extensively used in pharmacological and toxicological studies over the past few years. In this method, the liver is discrete from other organs by preserving the structure, function, and vascular system. Penetration occurs physiologically through the cells as well as the enzymatic pathways so that the mechanisms of metabolism and excretion are all naturally preserved (28). One of the most important advantages of this method is the relatively constant hepatic blood flow, which leads to minimal observed changes in the distribution and metabolism of various drugs due to the dependence of these processes on hepatic blood flow. 

Finally, in this research, the possible effects of untreated diabetes and post-treatment effects of metformin and/or cinnamon consumption on CYP2C19 were examined in diabetic rat isolated perfused liver model by measuring changes in the 5-hydroxy omeprazole-to-omeprazole ratio as an accepted probe for evaluating the CYP2C19 activity (29, 30).

## Materials and Methods

***Materials***

Pure racemic omeprazole was kindly supplied by TEMAD^®^ (Tehran, Iran). Nicotinamide was purchased from Merck (Darmstadt, Germany). Streptozotocin injectable solution was bought from Sizdah-e Aban Pharmacy (Tehran, Iran). Cinnamon bark extract powder was obtained from Roha Arzneimittel GmbH (Bremen, Germany). Metformin was purchased from Sigma Aldrich (St. Louis, MO, USA). Water used in all parts of the study was purified by Direct-W^®^ system (Millipore, France). Other chemicals that were used for the isolated hepatic perfusion study or HPLC analysis were of analytical reagent grades and obtained commercially.

***Animals***

Healthy male Wistar rats (age: 7 weeks, weight: 200-250 g) were randomly included in this assessment. The rats were kept under standard condition (according to IACUC), artificial light on a 12 hr light/dark cycle with free access to standard laboratory chow and water. 

***Induction of type II diabetes (T2DM)***

To induce diabetes, after 12 hr of fasting, 110 mg/kg Nicotinamide (31) (dissolved in normal saline) was intraperitoneally injected followed by the intraperitoneal injection of 65 mg/kg streptozotocin (dissolved in citrate buffer; pH = 4.5) 15 mins afterward. One week following diabetes induction, rats with blood sugar levels above 200 mg/dl were considered diabetic.

***Experimental design***

To investigate the effects of untreated diabetes and post-treatment effects of metformin and/or cinnamon consumption on the CYP2C19 activity, twenty-eight rats were divided into seven groups as follows:

1. The control group (i.e., healthy rats without diabetes induction) 

2. Diabetic rats receiving no treatment

3. Diabetic rats receiving only metformin treatment 

4. Normal rats receiving only metformin treatment 

5. Diabetic rats receiving both metformin and cinnamon treatment

6. Diabetic rats receiving only cinnamon treatment

7. Normal rats receiving only cinnamon treatment

Rats in the first two groups were administered 4 ml of normal saline by oral gavage once daily for two weeks. In metformin-receiving rats (i.e., rats in third, fourth, and fifth groups), 200 mg/kg of metformin (dissolved in 4 ml normal saline) was orally administrated once daily for two weeks following diabetes induction. Cinnamon-receiving rats (i.e., rats in fifth, sixth, and seventh groups) were administered 300 mg/kg of cinnamon (dissolved in 4 ml normal saline) by oral gavage for two weeks. The protocol was approved by the Institutional Review Board of Pharmaceutical Research Centre, Tehran University of Medical Sciences, Tehran, Iran. The ethical approval code number was [IR.TUMS.PSRC.REC.1396.3038].

***Surgical techniques***

Rats were anesthetized with an intraperitoneal injection of 75 mg/kg of ketamine and 15 mg/kg of xylazine solutions. The portal vein and inferior vena cava were catheterized by venous catheter (size: 16 and 18), and 500 units of heparin were injected into inferior vena cava. After inserting catheters, the liver was perfused with freshly prepared Krebs-Henseleit buffer (118 mM NaCl, 4.5 mM KCl, 2.75 mM CaCl_2_, 1.19 mM KH_2_PO_4_, 1.18 mM MgSO_4_, 25 mM NaHCO_3_ and 0.1 mM glucose), which was adjusted to the physiological pH (using 95% O_2_/ 5% CO_2_) for 10 min as an initial equilibration period.

***The isolated hepatic perfusion study***

After a washing period with blank fresh perfusate, a single-pass experiment was initiated with passing the omeprazole-containing medium (with inlet concentration of 400 μM) through the portal vein with a constant rate of 8.3 ml/min. Perfusate temperature (37±0.5 ^°^C) and pH (7.4±0.2) were monitored and kept within a constant range. Throughout the experiment, the pH level was adjusted by altering the inflow of carbogen if required. Liver transaminases activities (AST and ALT) were also continuously monitored by a spectrophotometric method used as a measure of liver viability. inferior vena cava was used as the outlet duct for sampling, and the perfusate samples (1 milliliter) were collected immediately after washing and then every 5 min in the first 30 mins and every 10 min in the second 30 mins and stored at -20 until analysis after centrifugation at 10000 rpm for 10 min. 

***Apparatus and chromatographic condition***

The chromatographic apparatus consisted of a low-pressure gradient HPLC pump coupled with a UV detector (302 nm), a 100 μlit loop, and a Rheodyne model 7725i injector, all from Knauer (Berlin, Germany). A Chromolith^TM^ Performance RP-18e 100 mm×4.6 column coupled with Chromolith^TM^ guard cartridge RP-18e 5 mm × 4.6 mm were applied for chromatographic separation (Merck, Darmstadt, Germany).

A mixture of 0.22 M Na_2_HPO_4_ buffer, adjusted to pH=5.9 using orthophosphoric acid, and acetonitrile (74:26, v/v) was employed as mobile phase and was passed through the system with a constant flow rate of 1 ml/min. Data acquisition and analyses were achieved by ChromGate chromatography software (Knauer, Berlin, Germany). Using this method, the retention time of 5-OH omeprazole and omeprazole were measured to be 3.5 and 12 min, respectively.

***Pharmacokinetic parameters***

Based on the concentrations of omeprazole and its metabolite, the areas under the concentration versus time curves AUC _(0-60)_ were acquired using the trapezoidal rule. The metabolic ratios at different times were calculated using metabolite concentration divided by parent drug concentration at each time point.

To determine mean concentration of omeprazole and its metabolite in a steady-state, the average concentration of each compound in perfusate at 40, 50, and 60 mins of liver perfusion were measured. 

***Statistical analysis***

All data in this study were expressed as mean±SEM. An unpaired t-test was employed in this study to determine differences between means of groups (*P*-value <0.05).

## Results

**Figure 1:**
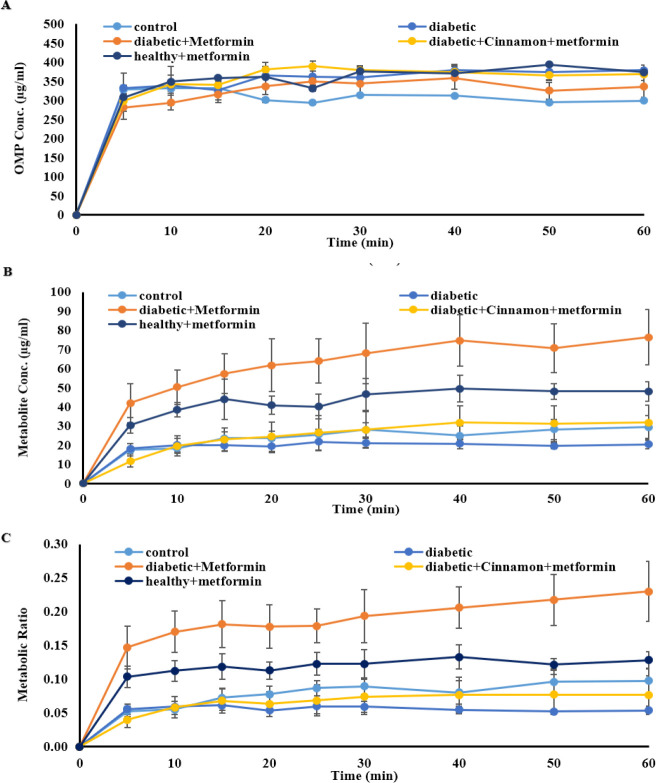
Perfusate concentration-time profile of omeprazole (A), 5-OH omeprazole (B) and mean metabolic ratio (C) in all metformin-receiving groups (2 weeks) compared to control and untreated diabetic rats (n=4 in each group, mean±SEM), following the passage of the perfusion buffer containing 400 μM omeprazole through the portal vein

**Figure 2:**
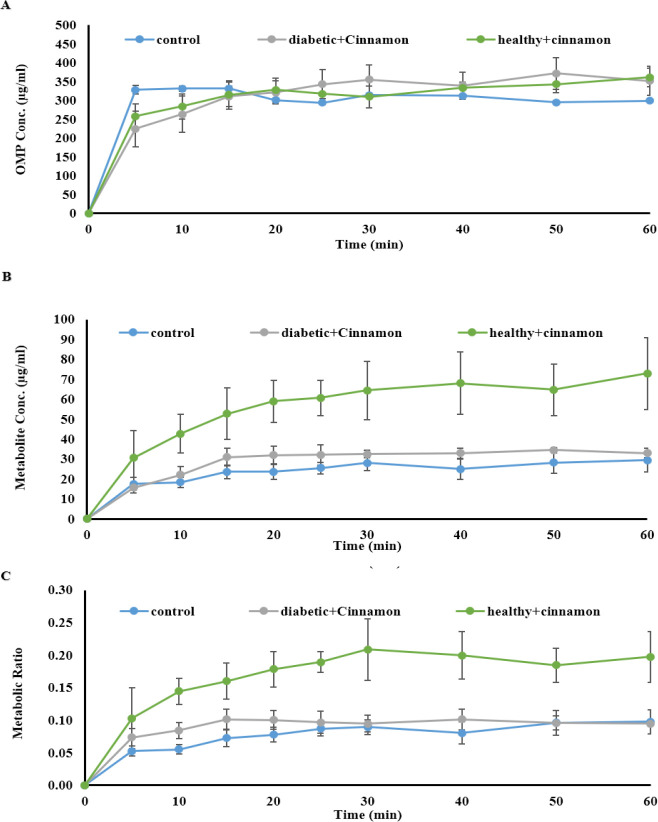
Perfusate concentration-time profile of omeprazole (A), 5-OH omeprazole (B) and mean metabolic ratio (C) in all Cinnamon-receiving groups (2 weeks) (except rats that received metformin plus Cinnamon) compared to control rats (n=4 in each group, mean±SEM), following the passage of the perfusion buffer containing 400 μM omeprazole through the portal vein

Figure 1 depicts the average perfusate concentration-time profiles of omeprazole (A), 5-OH omeprazole (B), and the corresponding metabolic ratios (C) in all metformin-receiving rats compared to the control and untreated diabetic rats. The mean perfusate concentration-time profiles of omeprazole (A), 5-OH omeprazole (B), and corresponding metabolic ratios (C) in all cinnamon-receiving groups (except for rats that received metformin plus cinnamon) compared to the control are demonstrated in Figure 2.

***The effect of T2DM on CYP2C19 activity***

**Figure 3:**
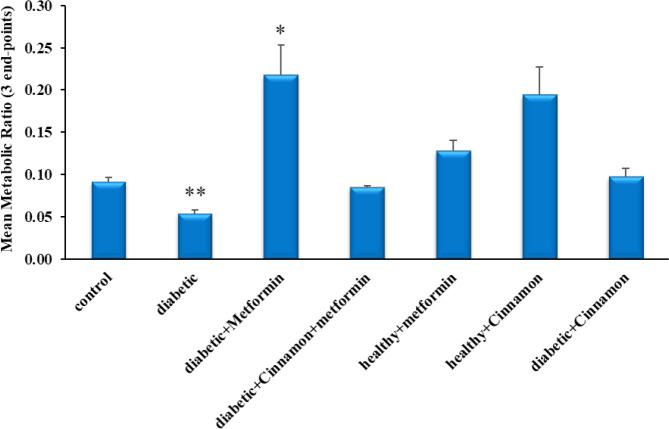
Perfusate means metabolic ratio profile (3 end-points) in all six groups compared to control rats, following the passage of the perfusion buffer containing 400 μM omeprazole through the portal vein (n=28, mean±SEM). Each experiment was repeated independently three times in triplicate tests, and data are shown as mean±SEM. *P≤ 0.05 compared to the control group

**Figure 4:**
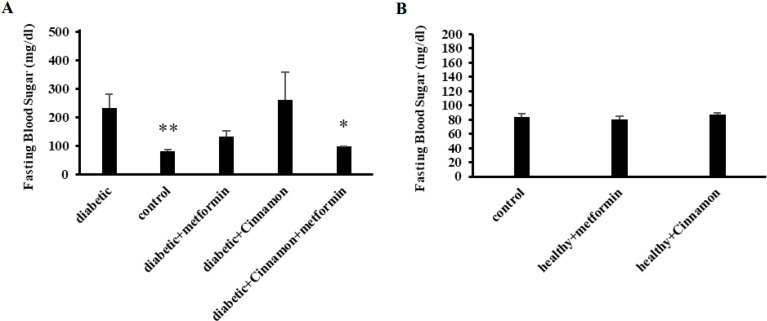
Fasting blood sugar (mg/dl) (FBS) of the control group and treated rats (with metformin or Cinnamon or metformin plus Cinnamon) in comparison with untreated diabetic rats (A). FBS of healthy rats that received metformin or Cinnamon compared to control rats (B). Each experiment was repeated independently three times in triplicate tests, and data are shown as mean±SEM. *P≤0.05 **P≤0.01 compared to the control group

The result of this research indicated that diabetes could significantly reduce the activity of CYP2C19 (by ~50%) since the mean metabolic ratios of omeprazole was changed from 0.091±0.005 in the control group to 0.054±0.005 in the untreated diabetic rats (*P*-value=0.003) (Figure 3). Predictably, fasting blood sugar (FBS) was significantly (*P*-value=0.001) higher in diabetic rats (233±49) than the control group (84±4) (Figure 4A).

***The effect of metformin consumption on the CYP2C19 activity in T2DM rats***

A significant increase (*P*-value=0.004) in enzyme activity following two weeks of metformin treatment was observed in the treated rats in comparison with the untreated diabetic rats since the metabolic ratios were changed from 0.054±0.005 in the untreated diabetic rats to 0.218±0.036 in the treated rats (Figure 3). Although FBS was decreased in the metformin-receiving diabetic rats (134±21) in comparison with the untreated rats (233±49), this alteration was not statistically significant (*P*-value=0.12) (Figure 4A). 

***The effect of metformin consumption on the CYP2C19 activity in healthy rats***

Since the level of enzyme activity following metformin treatment was also significantly higher than the control group (i.e., healthy rats), another group was added to the study to investigate the possible effects of metformin on increasing the enzyme activity in healthy rats.

Although increased enzyme activity was observed in the healthy rats receiving metformin (0.128±0.013) compared to the control group (0.091±0.005), the observed difference was not statistically significant (*P*-value=0.073) (Figure 3).

FBS of healthy rats receiving metformin (81±4) was not statistically different from the control group (84±4) (*P-value*=0.6)(Figure 4B), which is in accordance with other findings that hypoglycemia rarely happens in metformin consumption in non-diabetics.

***The effect of cinnamon consumption on the CYP2C19 activity in T2DM rats***

Results of this study indicated the modulating effect of cinnamon on the CYP2C19 activity in diabetic rats, so that despite its negligible impact on blood glucose levels (267±99) (*P*-value=0.83), cinnamon administration for 2 weeks led to returning enzyme activity (0.097±0.01) to the level observed in the control group (0.091±0.005) (*P*-value=0.67) (Figure 3). 

***The effect of cinnamon consumption on the CYP2C19 activity in normal rats***

Although an increase in mean metabolic ratios in healthy cinnamon-receiving rats was observed (0.194±0.049) in comparison with the control group (0.091±0.005), the observed difference was not statistically significant (*P*-value=0.058), which may be attributed to high inter-individual variations (Figure 3). 

Like rats in the healthy metformin-receiving group, blood glucose levels of rats in the healthy cinnamon-receiving group (87±2) were not significantly different from rats in the control group (84±4) (Figure 4B).

***The effect of simultaneous administration of cinnamon and metformin on the CYP2C19 activity in T2DM rats***

Despite an increase in enzyme activity following the consumption of metformin in diabetic rats (0.218±0.036) in comparison with the control group (0.091±0.005), co-administration of metformin and cinnamon in diabetic rats resulted in returning the activity of enzyme (0.085±0.002) to the observed level in the control group (0.091±0.005) (*P*-value=0.26) (Figure 3). Despite the lack of significant changes in FBS levels due to metformin (134±21) or cinnamon (267±99) consumption in diabetic rats, co-administration of cinnamon and metformin demonstrated significant (*P*-value = 0.03) FBS lowering effects on diabetic rats (99±1) in comparison with untreated diabetic rats (233±49) even with a short-course treatment (Figure 4A).

## Discussion

The activities of most drug-metabolizing enzymes (DMEs) are affected by genetic polymorphisms, and some degree of functionally significant polymorphism is also shown in the population (11). The resulting inter-genotype variability in the pharmacokinetics of certain medicines and their metabolites is often believed to account for a substantial fraction of inter-individual variability in drug responses (32). Most studies have focused almost exclusively on the DMEs’ genotypes in the population to provide a specific and safe genotype-based dosing regimen. This is based on the hypothesis that genotype is predictive of DME phenotype, as well as the pharmacokinetics and clinical response of the drug (5). The emphasis on correlating the DME genotype of patients with clinical outcomes carries a significant risk, which needs to be addressed; for instance, the implications of phenoconversion in which an extensive metabolizer (EM) genotype has the DME phenotype of a poor metabolizer (PM). Various environmental factors such as some diseases, either acute or chronic, lead to inflammatory responses, and increased levels of some cytokines such as interleukin-1β (IL-1β), IL-6, and tumor necrosis factor alpha (TNF-α) can change the expression and thus alter the amount of some of CYPs and cause a genotype-phenotype mismatch in drugs metabolism processes (11, 33). In view of the foregoing, it could be assumed that as an underlying disease, diabetes acts as an important environmental factor affecting the activity of CYP450 enzymes and therefore leading to difficulty in predicting the potential response to metabolized drugs.

In addition, although pharmaceutical ability to inhibit/induce P450 enzymes is usually well-characterized during development processes, herbal supplements are not required to be evaluated in this regard, before entering the market. This concept would be more problematic during the simultaneous use of pharmaceutical and herbal products such as cinnamon, which can lead to new and unexpected drug interactions due to changes in drug metabolism (34). This study investigated the effect of T2DM on the activity of cytochrome 2C19 with respect to changes in the metabolic ratio of omeprazole as a probe of the enzyme (30, 35). Additionally, the effect of single and simultaneous administration of metformin, as a first-line treatment for T2DM, and cinnamon, as a recommended herbal supplement (25), on the activity of enzyme along with changes in blood sugar profile was investigated in the isolated perfused liver (36). The results demonstrated that diabetes could significantly decrease (about 50%) the activity of this cytochrome with respect to average metabolic ratios of three last sampling time-points. Although treatment with metformin (for two weeks) could not significantly reduce FBS levels compared to the untreated rats, the treatment increased the activity of the enzyme by four times of the activity observed in the untreated rats and approximately twice of the observed enzyme activity in the control (healthy) group. The insignificant reduction in FBS levels is consistent with the results of Eskens *et al.* (2013) who did not observe any significant changes in the FBS profile in non-insulin-dependent diabetic rats receiving metformin for two weeks, which may be attributed to a short-course treatment (37).

To better understand the cause of significant increases in the activity of enzymes observed after receiving treatment in treatment reconvening rats compared to healthy rats (approximately 2-fold), another group was added to this study to assess the possible effects of metformin on enzyme activity in non-diabetic rats. Thus, the same metformin dosage was administered to healthy rats for two weeks. Although the average metabolic ratios showed an increase compared to the control group, no significant difference was observed between the two groups. The observed rise in enzyme activity could be a consequence of a short-course treatment (two weeks) in both the diabetic and control groups; however, it returns to normal with the continuation of treatment over time. Moreover, it could be concluded that the effect of metformin on modulating the CYP2C19 enzyme activity without affecting the blood glucose in diabetic rats may be attributed to the inhibitory effect of metformin on some inflammatory cytokines such as TNF-α, IL-1β, and IL-6, which is allegedly one of the main reasons for suppressing the CYP450 enzyme activity (13, 38). The effect of metformin on the mentioned cytokines has been reported by others (39). The results of this study indicate that the reduced cytochrome 2C19 activities due to diabetes possibly occur in patients, confirming the “phenoconversion” hypothesis associated with inflammatory diseases and making it necessary to consider the level of metabolic activity of the enzymes, especially in drug prescription associated with this enzyme system, such as clopidogrel. Additionally, the observed post-treatment return in enzymatic activity is another reason for the importance of continuous monitoring of the enzyme activity in these patients to increase therapeutic effects and reduce drug interactions.

Another objective of this study was to investigate the effect of cinnamon consumption on the cytochrome 2C19 activity. A considerable result of this study was the balancing effect of cinnamon powder on the activity of cytochrome 2C19 in T2DM; despite reduced enzyme activities resulting from diabetes compared to the control group, cinnamon consumption (two weeks) resulted in returning the levels of enzyme activity to the control group. Despite a significant return in the level of activity of the enzyme following cinnamon administration compared to the diabetic rats, blood glucose levels did not show any significant differences in comparison with the untreated diabetic rats. The lack of hypoglycemic effects of cinnamon in this study is consistent with Ranasinghe *et al.* (2012) who observed a significant change in FBS profile of diabetic rats following 20 days of cinnamon consumption; however, he did not observe a significant difference in first 10 days of treatment (40).

Along with the potential anti-inflammatory effects of metformin that was discussed above, there are numerous studies that show the inhibitory effects of cinnamon on the inflammatory and oxidative stress systems (41-43), which may explain the observed modulating impact of cinnamon on the CYP2C19 enzyme activity regardless of blood sugar levels.

Surprisingly, the return of enzyme activity to that of healthy rats (i.e., control group) was observed following the simultaneous administration of cinnamon and metformin in diabetic rats. No significant changes in FBS levels were observed in both metformin- and cinnamon-receiving diabetic rats in comparison with untreated diabetic rats, and a statistically significant lowering effect of FBS levels was shown after the co-administration of metformin and cinnamon. Generally, it can be concluded that the simultaneous use of cinnamon and metformin in T2DM can help to improve the function of the CYP2C19 enzyme and the prediction of drug response in these patients. However, it is essential to evaluate this hypothesis in T2DM patients to assess this effect directly in humans.

## Conclusion

The results of this study revealed that although T2DM can suppress the activity of CYP2C19 in rats, treatment with metformin sharply increases the enzyme activity (approximately 4-fold). The simultaneous administration of cinnamon and metformin can modulate the function of CYP2C19 to the observed level in the control group (i.e., healthy rats) and makes it more predictable to treat diabetes mellitus and the levels of other drugs that this enzyme is involved in their metabolism.
